# Supplementary material for: Detection and Molecular Characterization of Two Gammaherpesviruses from Pantesco Breed Donkeys during an Outbreak of Mild Respiratory Disease
Source: Viruses. 2021 Aug 2;13(8):1527. doi: 10.3390/v13081527 (PMC8402663; doi:10.3390/v13081527)
Supplement: Supplementary file 1 [file viruses-13-01527-s001.zip › Supplementary Figure S1.pdf]

Supplementary Figure S1. Phylogenetic trees of gammaherpesviruses identified in donkeys. Maximum likelihood trees based on the glycoprotein B (gB), terminase (ter), and DNA polymerase (DPOL) genes are shown. The trees were built with MEGAX with partial nucleotide sequences (gB: 405nt, ter: 356nt, DPOL: 182nt) using the maximum likelihood approach with Kimura 2 parameters (gB) or Tamura 3 parameters (DPOL and ter) models, identified as the best fitting models using the modeltest analysis implemented in MEGAX. A discrete Gamma distribution was used to model evolutionary rate differences among sites (+G = 0.4575 for gB, +G = 1.0948for DPOL, +G = 0.5394 for ter) and branch robustness was evaluated through 1000 bootstrap replicates. Species official (italics) or provisory names are indicated when available and sequences obtained in this study are indicated by the name of the donkey while the type of sample is specified in parenthesis.

gB

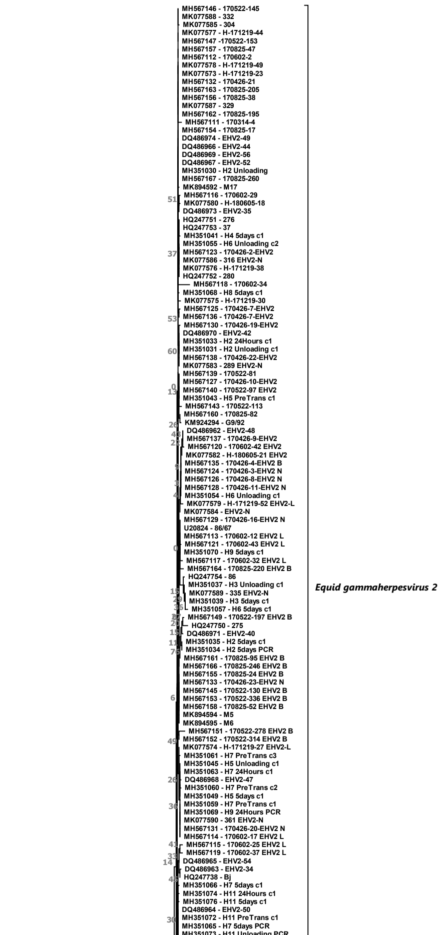
Equid gammaherpesvirus 2
Equid gammaherpesvirus 7
Asinine herpesvirus 5
Equid gammaherpesvirus 5
0.10

DPOL

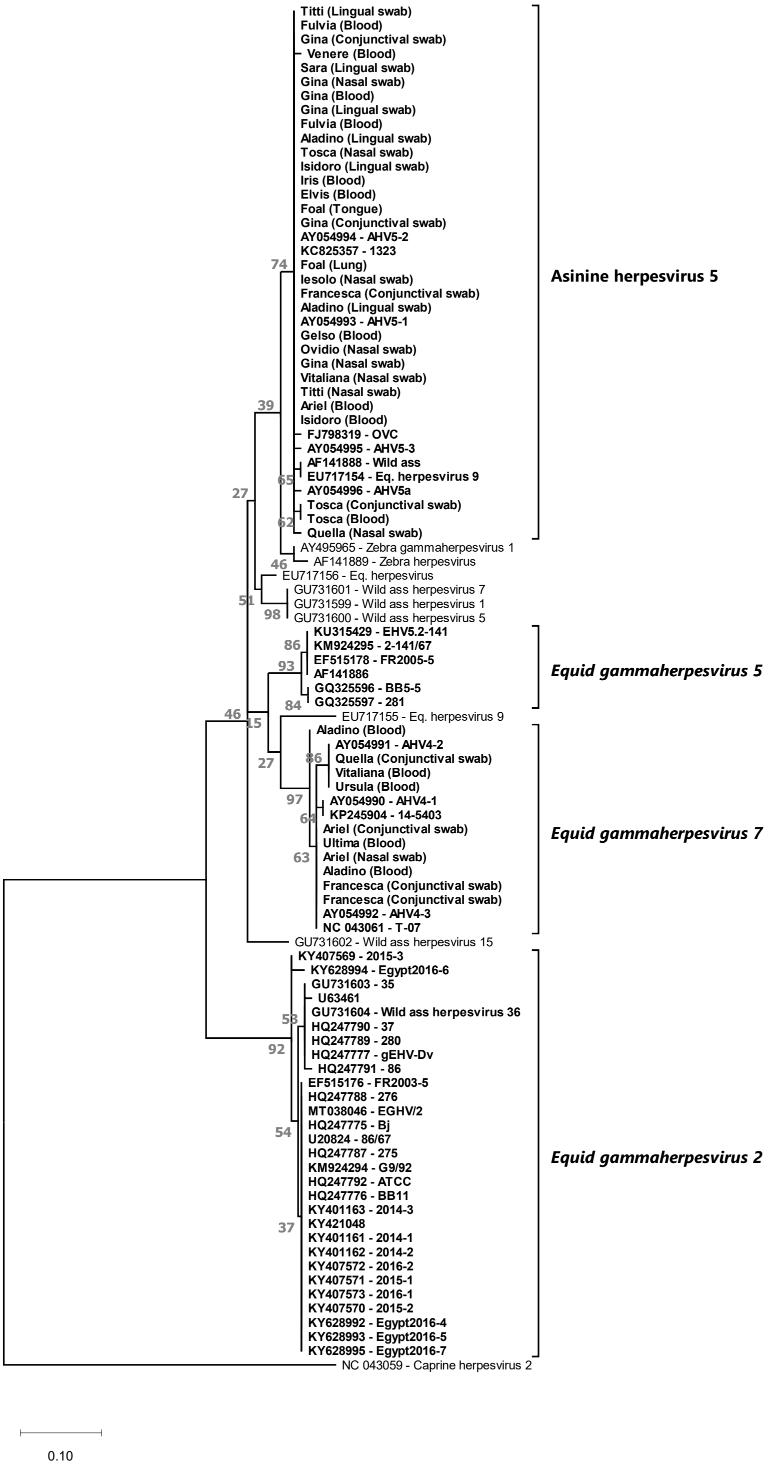
Titti (Lingual swab)
Fulvia (Blood)
Gina (Conjunctival swab)
Venere (Blood)
Sara (Lingual swab)
Gina (Nasal swab)
Gina (Blood)
Gina (Lingual swab)
Fulvia (Blood)
Aladino (Lingual swab)
Tosca (Nasal swab)
Isidoro (Lingual swab)
Iris (Blood)
Elvis (Blood)
Foal (Tongue)
Gina (Conjunctival swab)
AY054994 - AHV5-2
KC825357 - 1323
Foal (Lung)
Iesolo (Nasal swab)
Francesca (Conjunctival swab)
Aladino (Lingual swab)
AY054993 - AHV5-1
Gelso (Blood)
Ovidio (Nasal swab)
Gina (Nasal swab)
Vitaliana (Nasal swab)
Titti (Nasal swab)
Ariel (Blood)
Isidoro (Blood)
FJ798319 - OVC
AY054995 - AHV5-3
AF141888 - Wild ass
EU717154 - Eq. herpesvirus 9
AY054996 - AHV5a
Tosca (Conjunctival swab)
Tosca (Blood)
Quella (Nasal swab)
AY495965 - Zebra gammaherpesvirus 1
AF141889 - Zebra herpesvirus
EU717156 - Eq. herpesvirus
GU731601 - Wild ass herpesvirus 7
GU731599 - Wild ass herpesvirus 1
GU731600 - Wild ass herpesvirus 5
KU315429 - EHV5.2-141
KM924295 - 2-141/67
EF515178 - FR2005-5
AF141886
GQ325596 - BB5-5
GQ325597 - 281
EU717155 - Eq. herpesvirus 9
Aladino (Blood)
AY054991 - AHV4-2
Quella (Conjunctival swab)
Vitaliana (Blood)
Ursula (Blood)
AY054990 - AHV4-1
KP245904 - 14-5403
Ariel (Conjunctival swab)
Ultima (Blood)
Ariel (Nasal swab)
Aladino (Blood)
Francesca (Conjunctival swab)
Francesca (Conjunctival swab)
AY054992 - AHV4-3
NC 043061 - T-07
GU731602 - Wild ass herpesvirus 15
KY407569 - 2015-3
KY628994 - Egypt2016-6
GU731603 - 35
U63461
GU731604 - Wild ass herpesvirus 36
HQ247790 - 37
HQ247789 - 280
HQ247777 - gEHV-Dv
HQ247791 - 86
EF515176 - FR2003-5
HQ247788 - 276
MT038046 - EGHV/2
HQ247775 - Bj
U20824 - 86/67
HQ247787 - 275
KM924294 - G9/92
HQ247792 - ATCC
HQ247776 - BB11
KY401163 - 2014-3
KY421048
KY401161 - 2014-1
KY401162 - 2014-2
KY407572 - 2016-2
KY407571 - 2015-1
KY407573 - 2016-1
KY407570 - 2015-2
KY628992 - Egypt2016-4
KY628993 - Egypt2016-5
KY628995 - Egypt2016-7
NC 043059 - Caprine herpesvirus 2
Asinine herpesvirus 5
Equid gammaherpesvirus 5
Equid gammaherpesvirus 7
Equid gammaherpesvirus 2
0.10

ter

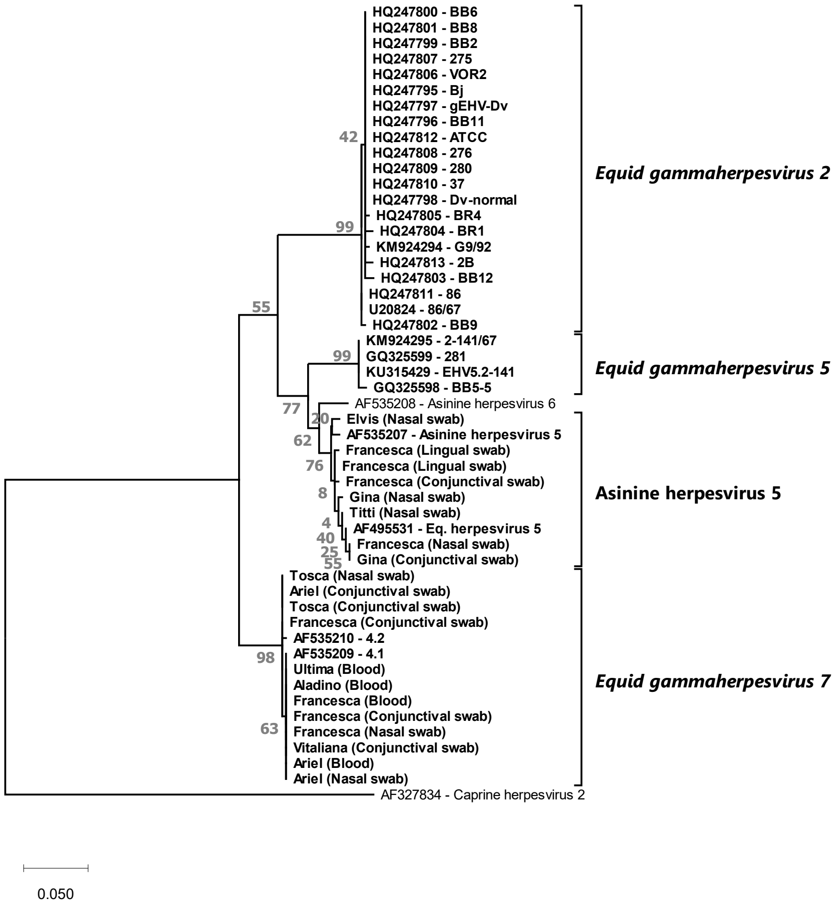
HQ247800 - BB6
HQ247801 - BB8
HQ247799 - BB2
HQ247807 - 275
HQ247806 - VOR2
HQ247795 - Bj
HQ247797 - gEHV-Dv
HQ247796 - BB11
HQ247812 - ATCC
HQ247808 - 276
HQ247809 - 280
HQ247810 - 37
HQ247798 - Dv-normal
HQ247805 - BR4
HQ247804 - BR1
KM924294 - G9/92
HQ247813 - 2B
HQ247803 - BB12
HQ247811 - 86
U20824 - 86/67
HQ247802 - BB9
KM924295 - 2-141/67
GQ325599 - 281
KU315429 - EHV5.2-141
GQ325598 - BB5-5
AF535208 - Asinine herpesvirus 6
Elvis (Nasal swab)
AF535207 - Asinine herpesvirus 5
Francesca (Lingual swab)
Francesca (Lingual swab)
Francesca (Conjunctival swab)
Gina (Nasal swab)
Titti (Nasal swab)
AF495531 - Eq. herpesvirus 5
Francesca (Nasal swab)
Gina (Conjunctival swab)
Tosca (Nasal swab)
Ariel (Conjunctival swab)
Tosca (Conjunctival swab)
Francesca (Conjunctival swab)
AF535210 - 4.2
AF535209 - 4.1
Ultima (Blood)
Aladino (Blood)
Francesca (Blood)
Francesca (Conjunctival swab)
Francesca (Nasal swab)
Vitaliana (Conjunctival swab)
Ariel (Blood)
Ariel (Nasal swab)
AF327834 - Caprine herpesvirus 2
42
99
55
99
77
20
62
76
8
4
40
25
55
98
63
Equid gammaherpesvirus 2
Equid gammaherpesvirus 5
Asinine herpesvirus 5
Equid gammaherpesvirus 7
0.050
