# Supplementary material for: Detection and Molecular Characterization of Two Gammaherpesviruses from Pantesco Breed Donkeys during an Outbreak of Mild Respiratory Disease
Source: Viruses. 2021 Aug 2;13(8):1527. doi: 10.3390/v13081527 (PMC8402663; doi:10.3390/v13081527)
Supplement: Supplementary file 1 [file viruses-13-01527-s001.zip › Supplementary Table S1.pdf]

Supplementary Table S1. Details of collected samples (bolded are positive samples).

	Id.	Oct 1	Oct 17/19	Nov 7
1	Aladino		**Blood-EDTA**<br>**Oral swab**	**Blood-EDTA**
2	Ariel		**Blood-EDTA**	**Blood-EDTA**<br>**Nasal swab**<br>**Conjunctival swab**
3	Dorotea		Blood-EDTA	Blood-EDTA
4	Elisabetta		Blood-EDTA	Blood-EDTA
5	Elvis		**Blood-EDTA**	**Blood-EDTA**<br>**Nasal swab**
6	Emma		Blood-EDTA	Blood-EDTA
7	Foal	**Tongue**<br>**Lung**<br>Intestine<br>Mesenteric LN<br>Spleen<br>Kidney		
8	Francesca		Blood-EDTA<br>**Nasal swab**<br>**Conjunctival swab**<br>**Tongue swab 1**<br>**Tongue swab 2**	**Blood-EDTA**<br>**Nasal swab**<br>**Conjunctival swab**
9	Fulvia		**Blood-EDTA**	**Blood-EDTA**
10	Gelso		**Blood-EDTA**	**Blood-EDTA**
11	Gina		Blood-EDTA<br>**Oral swab**<br>**Nasal swab**<br>**Conjunctival swab**	**Blood-EDTA**<br>**Nasal swab**<br>**Conjunctival swab**
12	Iesolo		Blood-EDTA	Blood-EDTA<br>**Nasal swab**
13	Iris		Blood-EDTA	**Blood-EDTA**
14	Isidoro		Blood-EDTA<br>**Oral swab**	**Blood-EDTA**<br>Nasal swab<br>Conjunctival swab
15	Laura		Blood-EDTA	Blood-EDTA
16	Mirella		Blood-EDTA	Blood-EDTA
17	Ornella		Blood-EDTA	Blood-EDTA<br>Nasal swab<br>Conjunctival swab
18	Ottavia		Blood-EDTA	**Blood-EDTA**
19	Ovidio		Blood-EDTA	Blood-EDTA<br>**Nasal swab**
20	Quella		Blood-EDTA<br>**Nasal swab**<br>Oral swab	Blood-EDTA<br>**Nasal swab**<br>**Conjunctival swab**
21	Quieta		Blood-EDTA	Blood-EDTA
22	Rossella		Blood-EDTA	Blood-EDTA
23	Sara		Blood-EDTA<br>**Oral swab**	Blood-EDTA<br>**Nasal swab**<br>**Conjunctival swab**
24	Serena		Blood-EDTA	
25	Temla		**Blood-EDTA**<br>**Oral swab**	Blood-EDTA<br>**Nasal swab**<br>**Conjunctival swab**

26	Titti	Blood-EDTA<br>**Oral swab**	Blood-EDTA<br>**Nasal swab**<br>**Conjunctival swab**
27	Tosca	**Blood-EDTA**<br>**Nasal swab**	Blood-EDTA<br>**Nasal swab**<br>**Conjunctival swab**
28	Ultima	Blood-EDTA	**Blood-EDTA**
29	Unica	**Blood-EDTA**	Blood-EDTA
30	Upupa	**Blood-EDTA**	Blood-EDTA
31	Ursula	Blood-EDTA	**Blood-EDTA**
32	Ustica	Blood-EDTA	Blood-EDTA
33	Valeria	**Blood-EDTA**<br>Oral swab	Blood-EDTA
34	Venere	**Blood-EDTA**	Blood-EDTA
35	Vinzia	Blood-EDTA	**Blood-EDTA**
36	Vitaliana	**Blood-EDTA**	**Blood-EDTA**<br>**Nasal swab**<br>**Conjunctival swab**
37	Zafira	Blood-EDTA	**Blood-EDTA**
38	Zaira	Blood-EDTA	Blood-EDTA
39	Zante	Blood-EDTA	**Blood-EDTA**
40	Zelante	**Blood-EDTA**	Blood-EDTA
