# Supplementary material for: Detection and Molecular Characterization of Two Gammaherpesviruses from Pantesco Breed Donkeys during an Outbreak of Mild Respiratory Disease
Source: Viruses. 2021 Aug 2;13(8):1527. doi: 10.3390/v13081527 (PMC8402663; doi:10.3390/v13081527)
Supplement: Supplementary file 1 [file viruses-13-01527-s001.zip › Supplementary Table S2.pdf]

Supplementary Table S2. Pairwise identity ranges within and between sequences of the two viral species obtained in this study.

	DPOL	gB	ter
Within *Equid gammaherpesvirus 7*	97.2-100%	99.3-100%	99.7-100%
Within Asinine herpesvirus 5	96.6-100%	93.3-100%	97.7-99.7%
Between the two species	84.1-88.3%	82-83%	90.9-91.9%
